# The decrease in panicle number is the main reason for the yield reduction of japonica rice caused by 1,2,4-trichlorobenzene stress

**DOI:** 10.3389/fpls.2024.1425945

**Published:** 2024-07-12

**Authors:** Feiyu Yan, Guoliang Zhang, Le Liu, Fang Wang, Hongliang Zhao, Zhiwei Huang, Yuan Niu

**Affiliations:** ^1^ School of Life Sciences and Food Engineering, Huaiyin Institute of Technology, Huai’an, China; ^2^ Jianghuai Plain Crop Industry Engineering Research Institute, Huaiyin Institute of Technology, Huai’an, China; ^3^ State Key Laboratory of Soil and Agricultural Sustainable Development, Nanjing Institute of Soil Research, Chinese Academy of Sciences, Nanjing, China

**Keywords:** japonica rice, yield, yield components, growth, 1,2,4-Trichlorobenzene

## Abstract

Persistent organic pollutants seriously affect the growth and development of crops. 1,2,4-Trichlorobenzene (TCB), as one of the most widely used chlorobenzenes, can affect the yield of japonica rice. However, existing research on the effect of TCB on japonica rice yield is not in-depth, and a basic understanding of commonality has not yet been formed. In this study, 28 conventional japonica rice varieties were selected to investigate the effects of TCB stress on their yield, yield composition, and TCB accumulation. This study also evaluated the efficiency of conventional tolerance indices in evaluating the TCB stress tolerance of japonica rice. The results showed that TCB caused sustained inhibition of the growth of japonica rice, which was considerably manifested in plant height, root length, soil plant analysis development (SPAD), and dry weight at different growth stages. Under TCB stress, TCB accumulation in various tissues of japonica rice increased sharply. TCB stress reduces the yield of japonica rice by reducing the number of panicles per hill, the number of spikelets per panicle, the grain filling percentage, and the grain weight. Overall, the results of this study indicate that TCB stress can cause a decrease in the yield of japonica rice, and the decrease in panicle number is the main reason. The conventional tolerance index can effectively evaluate the tolerance of japonica rice to TCB. The results of this study are substantial for the breeding and cultivation of japonica rice.

## 1 Introduction

Population increase and the rapid development of industry and agriculture have led to a substantial increase in the use of chemical materials, resulting in a high proportion of chemical substances being released into the air, soil, and water (Alexander, 1995). Persistent organic pollutants have received widespread attention because of their high toxicity, persistence, bioaccumulation, and long-distance transportation (Wang et al., 2005a). Excessive levels of persistent organic pollutants have been detected in soil and crops from multiple countries worldwide (Loganathan and Kannan, 1994; Gao et al., 2005). Chlorobenzenes, an important persistent organic pollutant, have several applications in agriculture; however, because of their hydrophobicity, chlorobenzenes can be absorbed by crops and accumulate in the food chain (Zhang et al., 2005). The environmental protection agencies of the United States and the European Community have included chlorobenzene in the priority control pollutant blacklist (Akinrinade et al., 2024). In addition, research has shown that the total chlorobenzene content in sludge from some cities in China ranges from 0.010 to 6.917 mg/kg (Cai et al., 2002). 1,2,4-Trichlorobenzene (TCB) is one of the most widely used chlorobenzenes in industrial applications, commonly used in the synthesis of insecticides. It is a colorless and low-molecular-weight hydrophobic organic compound with a logarithmic octanol/water partition coefficient of 4.1, which indicates high adsorption potential (Montgomery and Crompton, 2017; Dentel et al., 1998). With many TCBs entering the agricultural ecosystem, their impact on food security and agricultural production has caught attention (Ding et al., 2014).

Rice is a staple food for over half of the world’s population, and along with wheat and corn, it supplies 40% of the calories needed by humans. However, this number is higher in Asian countries where rice is the main staple (Gnanamanickam, 2009). The unique aquatic characteristics of rice make it a crop that consumes a huge amount of water, and water is the main pathway for the migration of organic pollutants. Therefore, rice is the food crop most vulnerable to the harm caused by organic pollutants (Akram et al., 2018). Moreover, TCB generated in industrialized areas can be deposited into the soil through atmospheric deposition, making it an important pollutant in the farmland of the Yangtze River Delta, one of the main japonica rice-producing areas in China (Pang et al., 2011). In these regions, the average chlorobenzene content in the cultivated layer of typical farmland is 35.53 ng/g (0.01–484.5 ng/g), with TCB contributing more than half of this content (Teng et al., 2008).

Previous studies have shown that TCB stress can affect the physiological and biochemical activities of rice, thereby affecting its growth and development (Niu et al., 2022). In terms of physiology and biochemistry, TCB stress can affect photosynthesis by reducing the maximum photosynthetic efficiency, actual photosynthetic efficiency, and RuBisCO enzyme activity of rice leaves (Wang et al., 2005b, Wang et al., 2006a). For respiratory function, TCB stress directly or indirectly inhibits the activity of amylase, cytochrome oxidase, and isoenzymes (Jin, 2003). In addition, TCB can cause oxidative damage in rice, and studies have shown that TCB stress increases the content of reactive oxygen species in rice leaves, leading to an increase in the accumulation of malondialdehyde. Furthermore, TCB stress induces antioxidant enzymes, enhancing their activity (Li et al., 2016). TCB stress also induces substantial upregulation of the expression levels of multiple metabolic node enzymes in rice leaves, including protein transglucosidase, inorganic pyrophosphorylase, and pyruvate orthophosphate double kinase (Ge et al., 2008). TCB can also cause damage to rice DNA, mainly manifested by a decrease in the DNA synthesis rate. In terms of growth and development, TCB stress can inhibit rice germination and reduce seedling height, root length, and biomass (Zhang et al., 2009). Further studies have shown that rice can transport TCB to stems, leaves, and grains through its roots, decreasing rice quality (He et al., 1996), mainly manifested by an increase in chalkiness and a decrease in the polished rice rate, whole-milled rice rate, and protein content. Moreover, the accumulation of TCB in the grains of indica rice is higher than that of japonica rice. TCB stress also reduces rice yield; however, agronomic measures such as increasing organic fertilizer can considerably reduce the toxic effect of TCB on rice, thereby increasing yield (Wang et al., 2006b).

As mentioned earlier, most of the existing research on the effects of TCB on rice has focused on physiological and molecular levels, with only a few studies exploring the effects of TCB on rice growth and yield. Rice is an important food crop, and achieving its high yield and stability is an ongoing pursuit. A few studies have reported that TCB stress can lead to reduced rice yield, but these studies are limited to one or a few varieties and have not delved into the reasons for yield reduction and forming a common understanding. Clarifying the effects of TCB on different yield components of rice and the inherent connections and interactions between these components under TCB stress, and proposing a suitable evaluation index for TCB tolerance in rice, has important guiding significance for rice breeding and cultivation. This study selected 28 common japonica rice varieties in the Yangtze River Delta region of China and systematically explored the effects of TCB stress on the yield and yield composition of japonica rice. The effectiveness of conventional tolerance indices in evaluating rice TCB stress was evaluated, providing theoretical guidance for rice breeding and cultivation under TCB stress.

## 2 Materials and methods

### 2.1 Experimental design and treatment

A pot experiment was conducted at the experimental site of the Huaiyin Institute of Technology. The test soil was collected from the experimental site with a pH of 7.12, organic matter content of 23.45 g/kg, total nitrogen content of 1.21 g/kg, available phosphorus content of 41.00 mg/kg, and available potassium content of 134.12 mg/kg. Two treatments were set up: adding 35 mg/kg TCB to the soil for TCB stress (TCB) and adding an equal amount of clean water to the control soil (CK). The diameter of the potted barrel was 25 cm above, 20.0 cm below, and 30 cm high. The application rates of fertilizers were N 2.0 g, K_2_O 1.8 g, and P_2_O_5_ 1.2 g per pot; the ratio of tillering fertilizer to panicle fertilizer was 5:5. Irrigation adopts a dry–wet alternation method. The potted area was equipped with a rain shelter to prevent the impact of rainwater on the TCB concentration of the soil. Bird nets were installed during maturity. **Table S1** lists the 28 conventional japonica rice varieties that were selected as plant materials. Rice seeds were disinfected with 0.5% sodium hypochlorite and washed with distilled water. After germination and sowing, seedlings with consistent growth were selected for transplantation. Each pot had three holes with one seedling per hill, and 60 pots were planted with rice for each variety. The cultivation management of rice was consistent with the local conventional cultivation management measures, and timely control of diseases and pests was performed.

### 2.2 Determination of growth-related indicators

Plant height, root length, dry weight, and soil plant analysis development (SPAD) values (using Konica Minolta SPAD-502Plus, Tokyo, Japan) of rice at the seedling, tillering, heading, and maturity stages were measured. Plant height and root length were measured using a graduated scale. For dry weight, an oven was used to dry the plant sample to a constant weight, and it was measured using an electronic balance. Nine rice plants were tested for each replicate (three replicates).

### 2.3 Determination of TCB content

Rice roots, the junction of roots and stems, stems, leaves, brown rice, and husks were obtained during the mature stage for TCB content determination. Three grams of freeze-dried samples crushed by a ball mill were obtained and mixed with 80 mL of 10% acetone *n*-hexane under ultrasound (25 W, 35°C) for 20 minutes for extraction. Subsequently, the mixture was centrifuged at 4,000 rpm for 5 minutes to extract the supernatant. The above extraction operation was repeated, merge the supernatant was merged and then evaporated, and the supernatant was concentrated. Afterward, a glass chromatography column filled with Na_2_SO_4_ and silica gel was used for further purification. The sample concentrate was rinsed with 50 mL *n*-hexane solvent, and the *n*-hexane rinse was collected and concentrated for gas chromatography analysis (Agilent 6820 gas chromatograph with ^63^Ni electron capture monitor; CD-5 capillary with a length of 30 m, an inner diameter of 0.25 mm, and a fixed phase film thickness of 0.25 μM). The carrier gas used was high-purity nitrogen (purity ≥ 99.999%). The injection port temperature was 250°C, with a programmed heating initial temperature of 50°C (stable for 3 minutes) and programmed heating at 10°C per minute to 160°C. The final temperature was 250°C at a rate of 30°C per minute, which was maintained for 2 minutes. The non-split injection volume was 1 μL. The detector temperature was 300°C (Langhorst and Nestrick, 1979).

### 2.4 Determination of yield and yield components

At the maturity stage of rice, 10 pots were taken from each variety to measure panicles per hill, grains per panicle, grain filling percentage, and grain weight and to calculate the yield. Before measuring all indicators, the sample was dried to a constant weight.

### 2.5 Tolerance index calculation

The stress susceptibility index (SSI), yield stability index (YSI), tolerance index (TOL), mean productivity (MP), geometric mean productivity (GMP), and stress tolerance index (STI) were calculated using the following formula (Bahrami et al., 2014).


SSI = (1−Ys/Yp)/(1−Yms/Ymp)

YSI = Ys/Yp

TOL = Yp−Ys

MP = (Yp + Ys)/2

GMP = (Yp*Ys) /2

$$STI\;=\;\left(Yp*Ys\right)/{\left(Ymp\right)}^{2}$$

where Ys and Yp are the yields of each genotype under TCB stress and non-stress conditions, respectively, and Yms and Ymp are the yield means for all genotypes under TCB stress and non-stress conditions, respectively.

### 2.6 Statistical analyses

All data were analyzed using SPSS software (Ver. 22.0, SPSS Inc., Chicago, IL, USA). ANOVA was applied to analyze the TCB accumulation data, and the mean differences were evaluated using Duncan’s multiple range test (p < 0.05). The Mann–Whitney–Wilcoxon test or paired t-test was used to analyze differences in other data, and Spearman’s correlation analysis was used to determine the correlation between various indicators. The figures were drawn using GraphPad Prism 8 (GraphPad Software Inc., San Diego, CA, USA).

## 3 Results

### 3.1 Growth of rice

**Figure 1:**
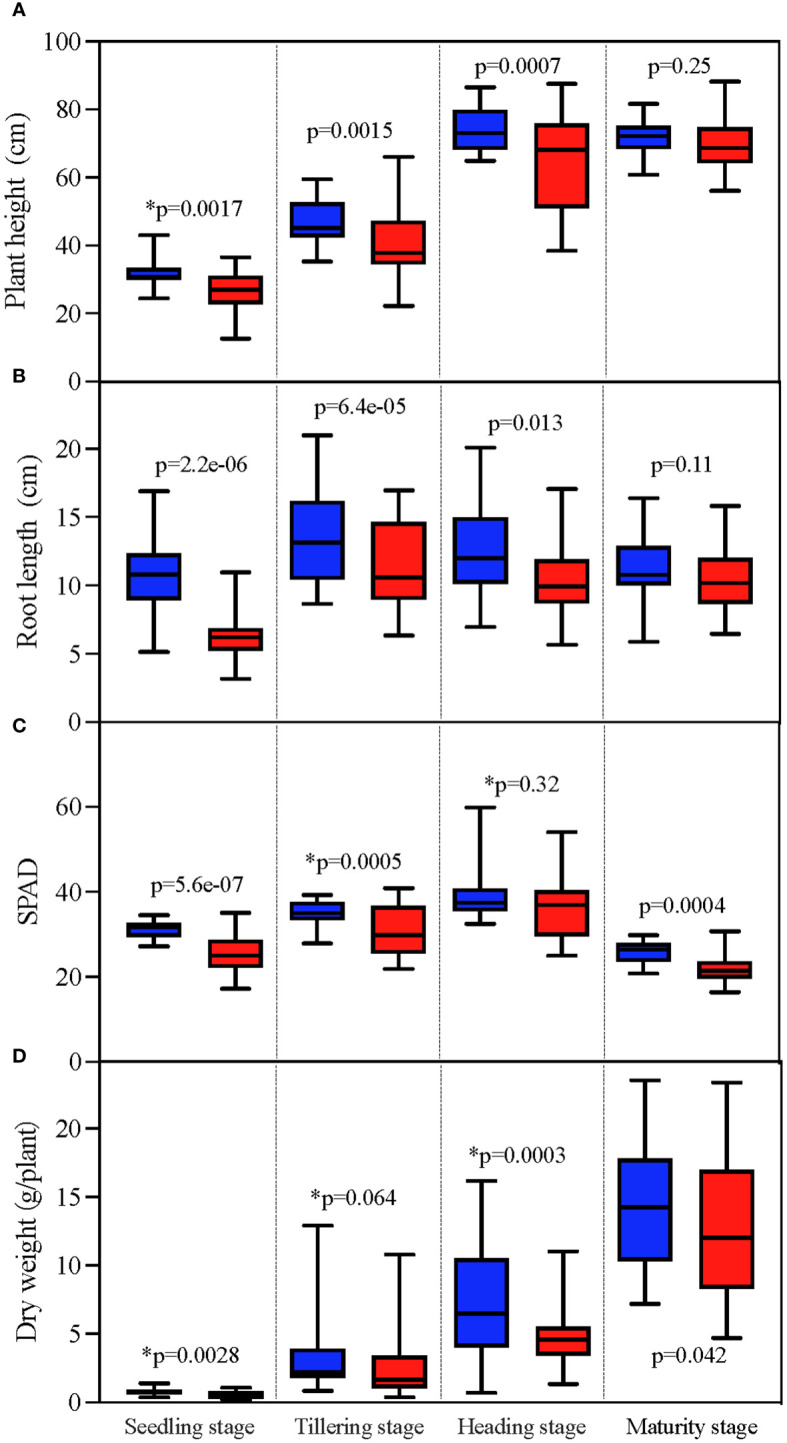
The effect of TCB stress on growth indicators of different growth stages of japonica rice. The unmarked p-value represents the result of the paired t-test, whereas the marked p-value with * represents the result of the Wilcoxon test. The red column represents indicators under TCB stress, whereas the blue column represents indicators under CK conditions. **(A)** Plant height. **(B)** Root length. **(C)** SPAD. **(D)** Dry weight. TCB, 1,2,4-trichlorobenzene; CK, control soil; SPAD, soil plant analysis development.

TCB has extensive and persistent inhibition on the growth of japonica rice, manifested in a decrease in plant height and root length, yellowing of leaves, and a decrease in dry weight. This inhibition is consistent in the seedling, tillering, heading, and mature stages. Substantial differences (p < 0.05) were observed in various indicators and periods between CK and TCB, except for plant height and root length in the mature stage (**Figure 1**).

### 3.2 TCB accumulation

**Figure 2:**
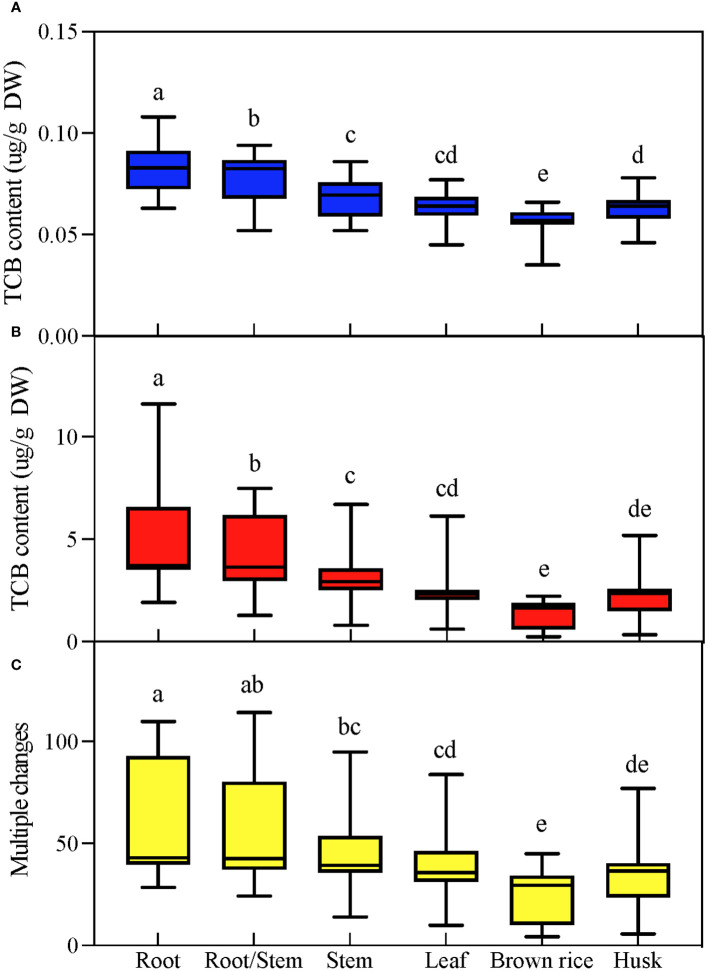
TCB content in various parts of mature japonica rice. According to Duncan’s multiple range test, the average value with different letters in each parameter was considerably different (p < 0.05). **(A)** TCB content under normal conditions. **(B)** TCB content under TCB stress conditions. **(C)** The multiple changes in tissue TCB content under TCB stress compared to normal conditions. TCB, 1,2,4-trichlorobenzene. Different letters in the data column indicate significant differences (p < 0.05) according to Duncan’s test.

Under CK conditions, each tissue of japonica rice contained trace amounts of TCB, with the highest TCB content in the root system and the lowest in brown rice. TCB stress greatly increased the TCB content in various tissues of japonica rice; however, the relative level of TCB content in each tissue was not affected by TCB stress, and the highest TCB content was still in the root system, while the lowest TCB content was in brown rice. By calculating the multiple changes in tissue TCB content under normal conditions corresponding to TCB stress, we found that the trend exhibited by these multiple changes is consistent with the tissue TCB content (**Figure 2**).

### 3.3 Yield and yield composition

**Figure 3:**
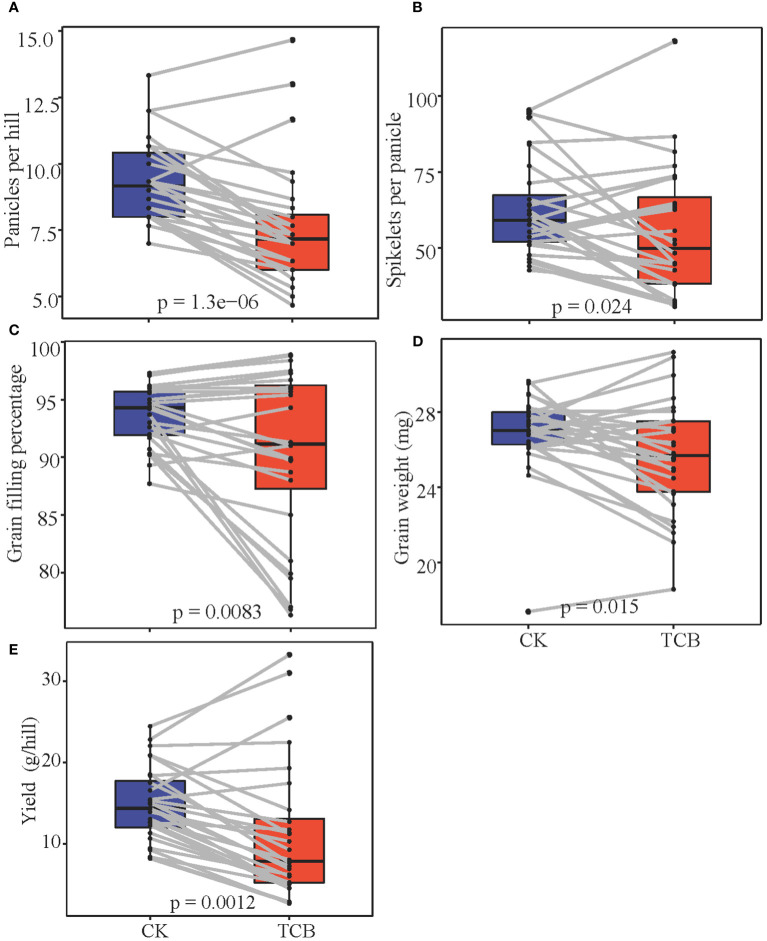
The effect of TCB stress on the yield and yield components of japonica rice. The p-value represents the result of the t-test. **(A)** Panicles per hill. **(B)** Grains per panicle. **(C)** Grain filling percentage. **(D)** Grain weight. **(E)** Yield. The line in the figure connects the indicator values of the same variety under normal conditions and TCB stress. TCB, 1,2,4-trichlorobenzene.

**Figure 4:**
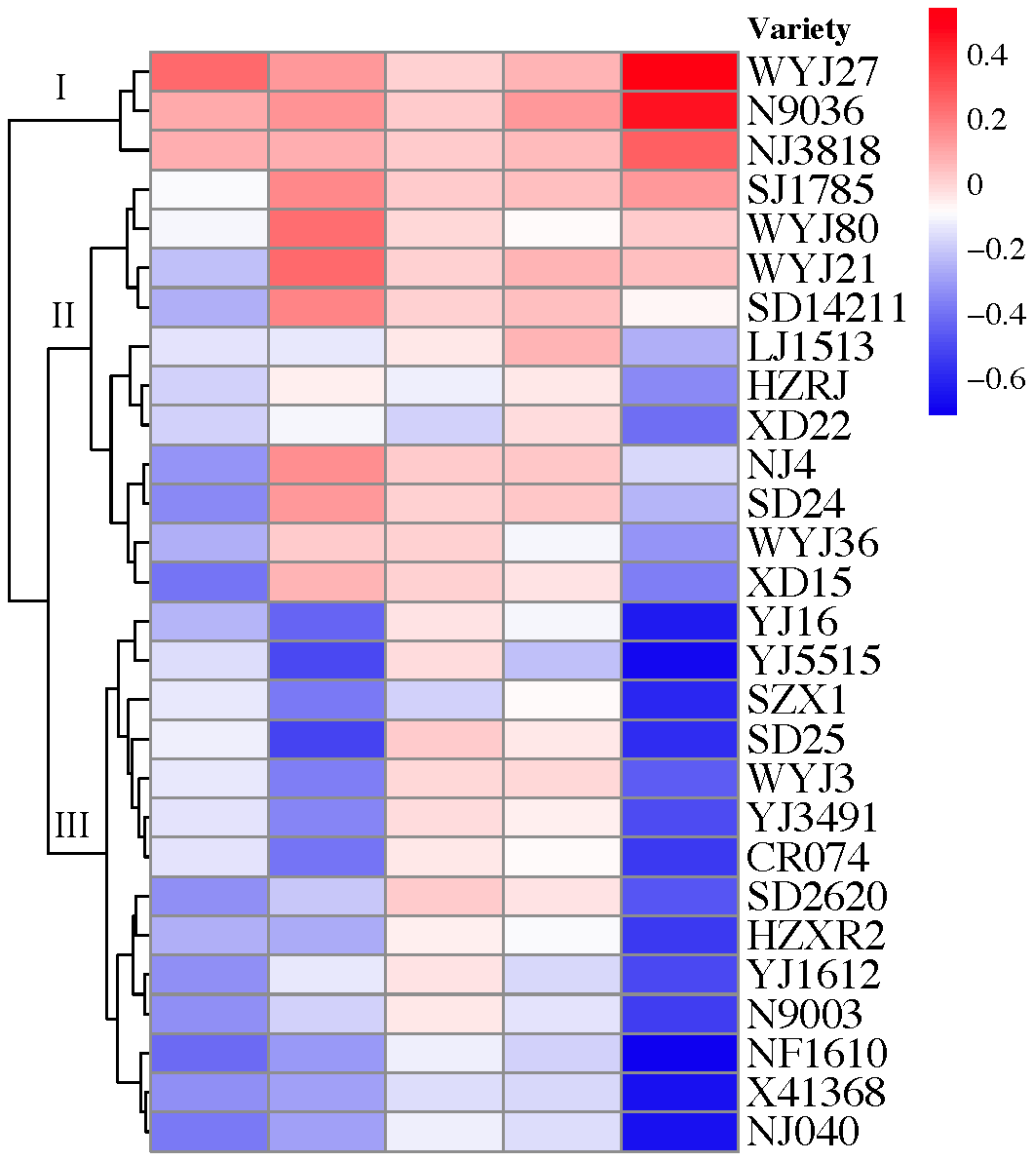
Cluster analysis of japonica rice based on yield and yield composition factors. The columns of color blocks from left to right represent the number of panicles per hill, grains per panicle, grain filling percentage, grain weight, and yield. The numerical quantification of color blocks represents the multiple changes of the indicator under TCB stress compared with CK conditions. The variety under branch I is yield-increasing type, the variety under branch II is yield-stable type, and the variety under branch III is yield-decreasing type. TCB, 1,2,4-trichlorobenzene.

TCB stress showed substantial (p < 0.05) inhibitory effects on the number of panicles per hill, grains per panicle, grain filling percentage, and grain weight of japonica rice. However, the yield and yield components of some varieties increased after exposure to TCB stress (**Figure 3**). As per the changes in yield and yield composition, the tested japonica rice varieties can be roughly divided into three categories: yield-increasing type, yield-stable type, and yield-decreasing type. Under TCB stress, the yield of the vast majority of rice varieties will decrease, with only a few varieties experiencing an increase in yield (**Figure 4**).

**Figure 5:**
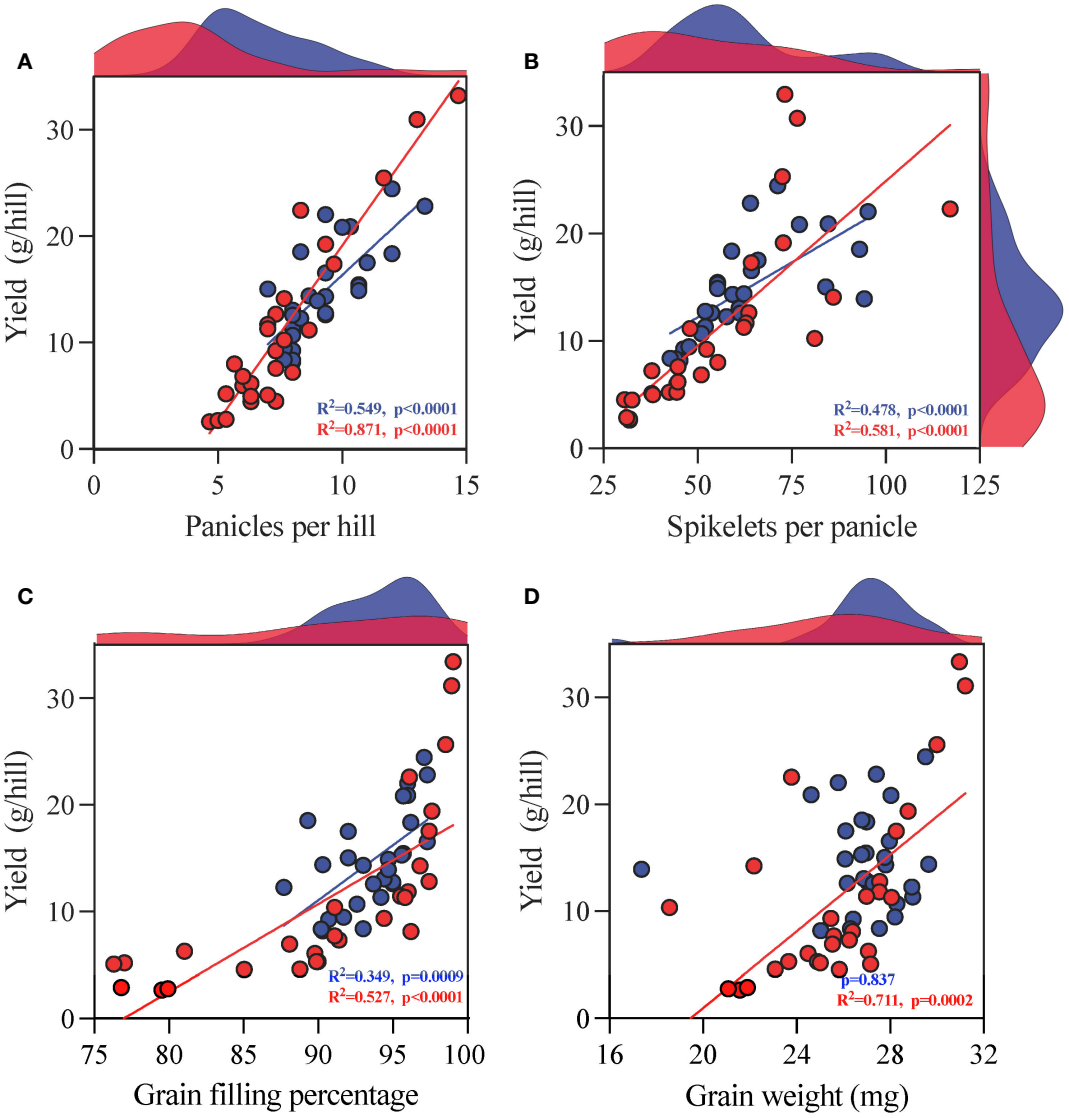
Regression analysis of yield components **(A)** panicles per hill, **(B)** grains per panicle, **(C)** grain filling percentage, and **(D)** grain weight] and yield of japonica rice. The red dots represent indicators under TCB stress, whereas the blue dots represent indicators under CK conditions. The red and blue patches on the graph represent the frequency distribution of the corresponding indicators on the horizontal or vertical axis under TCB stress and CK conditions, respectively. The red and blue lines represent the results of linear regression, whereas the red and blue data represent the R^2^ and p-values of linear regression, respectively. Non-substantial results are not labeled with R^2^.

The regression analysis results indicate that except for the lack of substantial correlation between grain weight and yield under CK conditions, all other indicators are considerably (p < 0.05) correlated under CK and TCB stress conditions. Moreover, TCB stress enhanced the impact of panicles per hill, grains per panicle, and grain weight on yield but reduced the impact of grain filling percentage on yield (**Figure 5**).

**Figure 6:**
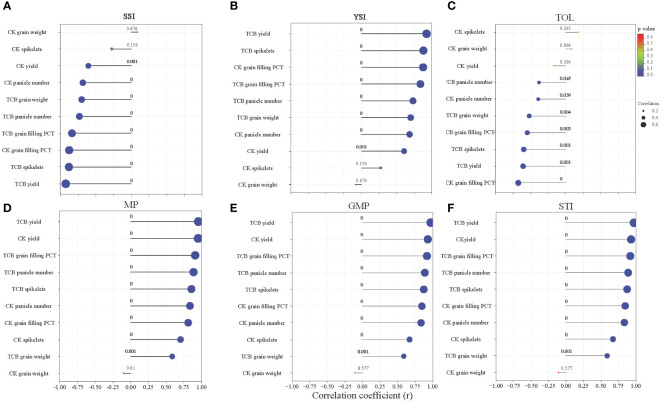
The correlation between yield and yield components of japonica rice and stress tolerance index: **(A)** SSI, **(B)** YSI, **(C)** TOL, **(D)** MP, **(E)** GMP, and **(F)** STI. The numbers in the figure represent the p-value, and the size of the dots represents the size of the correlation coefficient. SSI, stress susceptibility index; YSI, yield stability index; TOL, tolerance index; MP, mean productivity; GMP, geometric mean productivity; STI, stress tolerance index.

Most indicators of yield and yield components in japonica rice under CK and TCB stress conditions showed a substantial (p < 0.05) correlation with the tolerance index. The correlation coefficient between the same yield indicator and different tolerance indices varied greatly (**Figure 6**).

**Figure 7:**
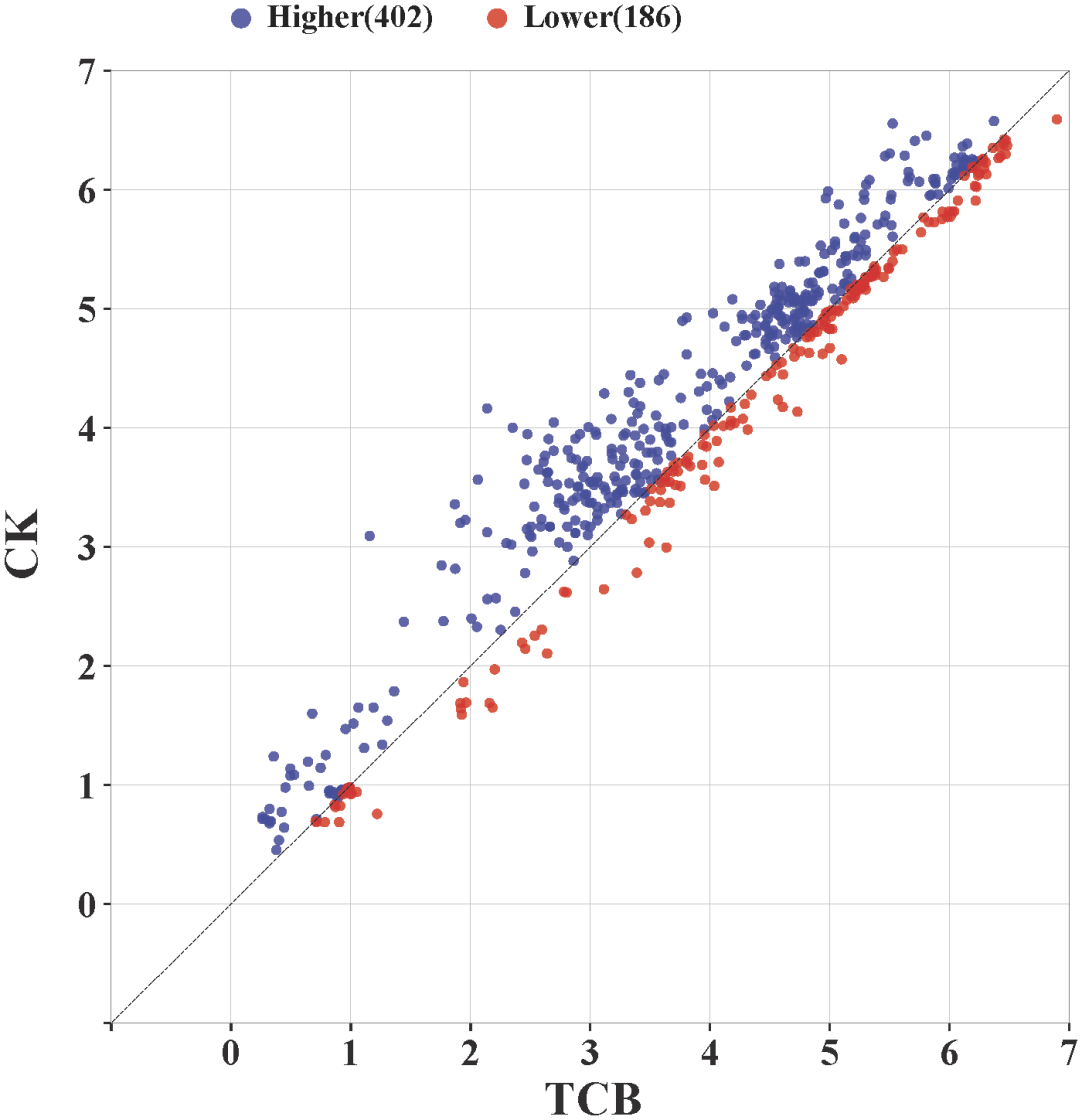
The effect of TCB stress on growth and yield-related indicators of japonica rice. The point in the graph is the logarithm of a variety’s indicator taken as base 2 under CK and TCB stress conditions and used as the horizontal and vertical coordinate values. The blue dots above the line represent a higher value of the indicator under CK conditions compared with TCB stress, whereas the red dots below the line represent a higher value of the indicator under TCB stress compared with CK conditions. TCB, 1,2,4-trichlorobenzene; CK, control soil.

By comparing the growth and yield-related indicators of all japonica rice varieties in this study, the results showed that under CK conditions, 402 data points were higher than those under TCB stress conditions, and 186 data points were lower than those under TCB stress conditions. Therefore, TCB has a broad inhibitory effect on the growth and yield of japonica rice (**Figure 7**).

## 4 Discussion

### 4.1 TCB stress inhibits the growth of japonica rice and leads to tissue TCB accumulation

Crop growth is inhibited under chlorobenzene stress conditions, and chlorobenzene accumulation is a common phenomenon (Wang and Jones, 1994b). Research has shown that TCB can reduce the dry weight and root length of vegetables (Song et al., 2012) and considerably inhibit the root system of beans (Liu et al., 2004). Monochlorobenzene, dichlorobenzene, and TCB all exhibit inhibitory effects on dry matter accumulation in maize with a dose–response inhibitory effect (Miguel et al., 2012). However, most of these studies only focus on a certain period of crop growth and development. In this study, we included the phenomenon of growth inhibition, and our findings show that TCB stress has a substantial inhibitory effect on the plant height, root length, SPAD, and dry weight of japonica rice (**Figure 1**). Notably, the inhibitory effect of TCB on japonica rice cannot be reduced or eliminated with its growth process. During the seedling, tillering, heading, and maturity stages, the growth-related indicators of japonica rice under TCB stress were considerably lower than those of the control. In a study using a TCB stress concentration of 20 mg/kg, its inhibitory effect on rice growth can be partially restored in the heading and booting stages, mainly manifested by the restoration of rice plant height and dry weight to the same level as under CK conditions during these growth stages (Wang, 2006). However, this is different from the results of our study. We speculate that this may be related to the TCB stress applied to the entire growth process of japonica rice and the high concentrations of TCB (35 mg/kg) as a stress in our study. There are also studies indicating that low concentrations of TCB can stimulate crop growth, manifested as an increase in root system and plant height. In addition, extensive research has found that chlorobenzenes accumulate in various crops. In a vegetable study conducted in Hangzhou, China, five vegetables were detected to contain various chlorobenzene substances, including TCB (Zhang et al., 2005). Similarly, nine types of vegetables in the UK have been detected to have accumulated chlorobenzenes (Wang and Jones, 1994a). In our study, we found that the root system of japonica rice is the tissue with the highest accumulation of TCB, whereas brown rice had the lowest, and this performance was consistent under normal and TCB stress conditions (**Figure 2**). Nevertheless, we believe that TCB stress can still lead to the problem of organic matter pollution in food. In another study, spinach roots have also been found to have the highest accumulation of chlorobenzenes in tissues (Zhang et al., 2005). This may be because the root system of crops is the first line of defense against stress damage, which, when faced with stress such as heavy metals and high salinity, is also the tissue with the most accumulation of harmful ions and with the clearest inhibition (Rodriguez-Serrano et al., 2006).

### 4.2 TCB stress reduces the yield of japonica rice

High yield and quality are both ongoing pursuits in crop production (Huang et al., 2017). In rice grown under optimal environmental conditions, the number of panicles per plant, grains per panicle, grain weight, and grain filling percentage determine its yield (Xing and Zhang, 2010). However, unfavorable pressure can lead to a decrease in rice yield and a deterioration in quality. Chlorobenzenes can seriously affect the yield of wheat and rice, and a similar phenomenon has also been observed in horticultural crops (Wang et al., 2006b; Li et al., 2016). A previous study classified rice into five categories based on its tolerance to TCB, with resistant varieties experiencing increased yield under low concentrations of TCB stress (Niu et al., 2023). In our study, we also found similar varieties in which one or more indicators, such as the number of panicles per hill, grains per panicle, grain filling percentage, and grain weight, are all promoted by TCB stress (**Figure 3**). Although the yield of these varieties has increased, their TCB content in brown rice has also increased, and the quality of rice has been negatively affected (**Figure 4**). Overall, the impact of TCB on the yield and yield composition of japonica rice is negative. After TCB stress, the number of panicles per hill, grains per panicle, grain filling percentage, and grain weight of japonica rice considerably decreased, leading to a substantial decrease in yield. In contrast, in an experiment using one variety of japonica rice and one variety of indica rice as test materials, TCB considerably reduced the number of panicles per pot, grains per panicle, and grain filling percentage but did not affect grain weight. The performance of indica and japonica rice is consistent (Wang et al., 2006b). We speculate that this is due to variety specificity, as we also observed in the experiment that the grain weight of some varieties was not inhibited by TCB. We also observed that TCB stress enhanced the effects of panicles per hill, grains per panicle, and grain weight on yield but reduced the effect of grain filling percentage on yield (**Figure 5**). Simultaneously, the number of panicles per hill was also most strongly inhibited by TCB (TCB stress decreased by 19.6% compared with CK), and the number of panicles per hill mainly depends on the tillering ability of japonica rice. However, during the tillering stage, TCB severely inhibits rice growth (**Figure 1**). These results indicate that a higher number of panicles per hill under TCB stress conditions may be the main trait for achieving high yield. In the growth and development cycle of rice, many factors can affect yield, among which the most important are light intensity, photosynthetic efficiency, nitrogen use efficiency, and water use efficiency (Nutan et al., 2020). Previous studies have shown that TCB stress reduces the leaf area index and water use efficiency of rice, which can lead to a decrease in yield. Water and nutrient deficiencies hinder leaf growth, thereby affecting the interception of light radiation by the leaves (Gardner et al., 1985; Wang et al., 2005b). Photosynthesis is the foundation of almost all life on Earth and a major component of crop yield (Gibson et al., 2011). Photosynthesis inhibited by TCB stress can also decrease the yield. Therefore, breeding high photosynthetic efficiency varieties is beneficial for achieving a high yield of japonica rice under TCB stress. Nitrogen is a key component of plant development and crop yield because of its impact on radiation use efficiency and photosynthesis (Sinclair and Horie, 1989). Research has shown that TCB can reduce the nitrogen utilization efficiency of rice, thereby affecting the source–sink characteristics of rice, leading to a reduced yield. Increasing nitrogen fertilizer application can replace the impact of TCB on rice yield (Wang et al., 2006b).

### 4.3 Comprehensive evaluation of the impact of TCB stress japonica rice yield and growth

Evaluating crop tolerance to adversity is a complex process. Different stress characteristics and growth stages can lead to different stress responses in the same crop (Bhatnagar-Mathur et al., 2008). Using yield to evaluate crop tolerance to adversity is an effective method. Our research results indicate that several stress tolerance indices correlate well with the yield and yield components of japonica rice, indicating that these stress tolerance indices can be used to evaluate the tolerance of japonica rice to TCB (**Figure 6**). The existing research on TCB stress in rice roughly divides rice into three categories—growth-promoting type, growth stable type, and growth-inhibiting type—which was also the basis of some studies that also classified rice into five categories based on this (Niu et al., 2022, Niu et al., 2023). However, previous studies included fewer rice varieties and did not focus on the entire growth period of rice. Our research on the full growth period of multiple varieties indicates that although the growth of some varieties is promoted by TCB, overall, TCB has caused extensive inhibition of the growth and yield indicators of japonica rice (**Figure 7**). How to improve the yield of japonica rice under TCB stress is still a topic worth studying.

## 5 Conclusions

TCB has caused sustained inhibition of the growth of japonica rice, manifested in substantial inhibition of plant height, root length, SPAD, and dry weight of japonica rice at different growth stages. The tissue with the highest accumulation of TCB in japonica rice under TCB stress is the root, and the tissue with the lowest accumulation is brown rice. TCB stress reduced the yield of japonica rice by reducing the number of panicles per hill, grains per panicle, grain filling percentage, and grain weight, with panicles per hill as the most strongly suppressed indicator. TCB causes extensive inhibition of the growth of japonica rice, and the use of tolerance indices can effectively evaluate the TCB tolerance of japonica rice.

## Data availability statement

The original contributions presented in the study are included in the article/**Supplementary Material.** Further inquiries can be directed to the corresponding author.

## Author contributions

FY: Formal analysis, Funding acquisition, Investigation, Methodology, Resources, Software, Validation, Visualization, Writing – original draft, Writing – review & editing. GZ: Conceptualization, Funding acquisition, Methodology, Resources, Software, Supervision, Writing – review & editing. LL: Data curation, Writing – review & editing. FW: Data curation, Writing – review & editing. HZ: Investigation, Writing – review & editing. ZH: Validation, Writing – review & editing. YN: Visualization, Writing – review & editing.
