# Supplementary material for: The decrease in panicle number is the main reason for the yield reduction of japonica rice caused by 1,2,4-trichlorobenzene stress
Source: Front Plant Sci. 2024 Jul 12;15:1425945. doi: 10.3389/fpls.2024.1425945 (PMC11272625; doi:10.3389/fpls.2024.1425945)
Supplement: Supplementary file 1 [file Table_1.docx]

Supplementary Material

The Decrease in Panicle Number is the Main Reason for the Yield Reduction of Japonica Rice Caused by 1,2,4-Trichlorobenzene Stress

**Feiyu Yan, Guoliang Zhang*, Le Liu, Fang Wang, Hongliang Zhao, Zhiwei Huang, Yuan Niu.**

*** Correspondence:** Guoliang Zhang; hgzgl@hyit.edu.cn

# Supplementary Tables

Table. S1 Information on the selected japonica rice varieties in this study

| **Chinese name** | **English name** | **Abbreviation** | **Variety characteristics** |
| --- | --- | --- | --- |
| 宁9036 | Ning9036 | N9036 |  |
| 南粳3818 | Nanjing3818 | NJ3818 | https://www.ricedata.cn/variety/varis/618046.htm |
| 武运粳27号 | Wuyunjing27 | WYJ27 | https://www.ricedata.cn/variety/varis/609151.htm |
| 武运粳21号 | Wuyunjing21 | WYJ21 | https://www.ricedata.cn/variety/varis/601962.htm |
| 苏粳1785 | Sujing1785 | SJ1785 |  |
| 宁粳4号 | Ningjing4 | NJ4 | https://www.ricedata.cn/variety/varis/605677.htm |
| 泗稻14-211 | Sidao14-211 | SD14211 |  |
| 圣稻24号 | Shengdao24 | SD24 | https://www.ricedata.cn/variety/varis/618129.htm |
| [徐稻15号](http://www.baidu.com/link?url=rIpQfQe-dfwcu_s_gLI8Ce71iFFQjS-_iAE6G7TRw0N5QFsOCBYDmqXLqLJmN5t52PvGZqNHfZEC5fpBtyifAK) | Xudao15 | XD15 | https://www.ricedata.cn/variety/varis/623412.htm |
| 沪早香软2号 | Huzaoxiangruan2 | HZXR2 | https://www.ricedata.cn/variety/varis/622600.htm |
| 宁9003 | Ning9003 | N9003 |  |
| 扬粳1612 | Yangjing1612 | YJ1612 |  |
| 南繁1610 | Nanfan1610 | NF1610 |  |
| 宁粳040 | Ningjing040 | NJ040 | https://www.ricedata.cn/variety/varis/604667.htm |
| 徐41368 | Xu41368 | X41368 |  |
| 圣稻2620 | Shengdao2620 | SD2620 |  |
| 武运粳80 | Wuyunjing80 | WYJ80 | https://www.ricedata.cn/variety/varis/616532.htm |
| 武育粳36号 | Wuyujing36 | WYJ36 |  |
| 武育粳3号 | Wuyujing3 | WYJ36 | https://www.ricedata.cn/variety/varis/601119.htm |
| 扬粳3491 | Yangjing3491 | YJ3491 | https://www.ricedata.cn/variety/varis/618049.htm |
| 圣稻25号 | Shengdao25 | SD25 | https://www.ricedata.cn/variety/varis/618132.htm |
| 扬粳5515 | Yangjing5515 | YJ5515 |  |
| 连粳1513 | Lianjing1513 | LJ1513 |  |
| 盐粳16 | Yanjing16 | YJ1612 | https://www.ricedata.cn/variety/varis/616202.htm |
| 沪早软粳 | Huzaoruanjing | HZRJ |  |
| 松早香1号 | Songzaoxiang1 | SZX1 | https://www.ricedata.cn/variety/varis/615081.htm |
| 新稻22 | Xindao22 | XD22 | https://www.ricedata.cn/variety/varis/609128.htm |
| 常软07-4 | Changruan07-4 | CR074 |  |
